# Targeting negative phosphorylation to activate AMPK

**DOI:** 10.1530/EC-25-0260

**Published:** 2025-07-17

**Authors:** Alexia Pearah, Balamurugan Ramatchandirin, Karina Ramirez, Sally Radovick, Fredric E Wondisford, Ling He

**Affiliations:** ^1^ Department of Pediatrics, Johns Hopkins University School of Medicine, Baltimore, Maryland, USA; ^2^ Department of Basic Medical Sciences, University of Arizona College of Medicine, Phoenix, Arizona, USA; ^3^ Department of Medicine, University of Arizona College of Medicine, Phoenix, Arizona, USA

**Keywords:** AMPK phosphorylation, targeting-peptide, gluconeogenic gene expression

## Abstract

AMPK is a master regulator of metabolism and is highly conserved and ubiquitously expressed. Activation of AMPK stimulates the catabolic pathway (glucose utilization and β-oxidation) and inhibits the anabolic pathway (gluconeogenesis, protein synthesis, and lipogenesis), leading to improvement of cellular energy status. However, the mechanisms of maintaining low cellular AMPK activity are not fully understood. We and other investigators showed that activated PKA in the glucagon-cAMP signaling pathway and insulin-activated AKT both can directly phosphorylate AMPKα1/2 at S496/491 to inhibit AMPK activity. In the current study, we found that activation of AMPK by an activator, AICAR, led to elevated and prolonged phosphorylation of AMPKα1/2 at S496/S491, reflecting a feedback inhibition of AMPK activity. In an *in vitro* assay, functional AMPKα1β1γ1 or AMPKα2β1γ1 can phosphorylate AMPKα1 at S496 or AMPKα2 at S491, respectively. We designed and successfully screened a new AMPKα2-targeting peptide to activate AMPK through competitively blocking the negative phosphorylation, resulting in suppression of gluconeogenic gene expression and promotion of mitochondrial fission in hepatocytes.

## Introduction

AMPK, a master regulator of metabolism, is highly conserved and ubiquitously expressed. Functional AMPK is a heterotrimeric complex, consisting of a catalytic α subunit, scaffold protein β subunit, and regulatory γ subunit; each subunit exists as multiple isoforms (α1, α2, β1, β2, γ1, γ2, γ3) and is encoded by separate genes (1, 2, 3). These isoforms of each subunit could give 12 different AMPK heterotrimeric combinations; each one may posit a distinct function (4). With decreasing cellular energy status, an elevated AMP/ATP or ADP/ATP ratio will augment AMP or ADP binding to the γ subunit, leading to an allosteric change in the AMPK complex to increase the phosphorylation of the α subunit at T172 either by an upstream kinase or by prevention of dephosphorylation by a protein phosphatase (5, 6, 7). The phosphorylation of the AMPKα subunit at T172 by upstream kinases could increase kinase activity by over 100-fold (5).

Activation of AMPK by antidiabetic drugs, such as metformin, can alleviate hyperglycemia in obesity and type 2 diabetes mellitus (T2DM) by suppressing liver glucose production (8, 9, 10). This is through stimulating the formation of a functional AMPKαβγ complex (11), subsequently increasing CBP phosphorylation at S436 and the disassembly of the ‘gluconeogenic engine’, the CREB-CBP/CRTC2 complex (12, 13). Activation of AMPK in muscle can increase glucose uptake and utilization (14, 15). Activated AMPK can directly phosphorylate ACC1/2 to reduce malonyl-CoA levels, thus inhibiting *de novo* lipogenesis (16). Reduction of malonyl-CoA levels can lead to the disinhibition of CPT1 and an increase in fatty acid oxidation in the mitochondria. In addition, activated AMPK also phosphorylates SREBP1, the master regulator in lipogenesis, to inhibit its function (17). Activated AMPK can inhibit protein synthesis through negative phosphorylation of TSC2 and raptor in the mTORC1 signaling pathway (18, 19). Therefore, activation of AMPK stimulates the catabolic pathway (glucose utilization and β-oxidation) and inhibits the anabolic pathway (gluconeogenesis, protein synthesis, and lipogenesis) (5, 20), leading to the alleviation of hyperglycemia and liver steatosis in obesity and T2DM (12, 21, 22, 23).

The molecular mechanism of maintaining AMPK at low activity levels is not fully understood. In the regulation of metabolism, insulin and glucagon antagonize each other’s activity. Our previous studies showed that activated PKA in the glucagon-cAMP-PKA signaling can directly phosphorylate AMPKα1 (the principal AMPK catalytic subunit in the liver (8)) at S496 (previously designated as S485) to impair AMPK activity in hepatocytes (22, 24). However, insulin-activated AKT can also directly phosphorylate AMPKα1 at S496 to inhibit AMPK activity (25, 26, 27). Thus, glucagon-activated PKA and insulin-activated AKT can work together to inhibit AMPK activity. Even though AMPK is a promising therapeutic target for treating obesity and T2DM, to date, there is no direct AMPK activator clinically available.

## Materials and methods

### Animal experiments

All animal protocols were approved by the Institutional Animal Care and Use Committee of the University of Arizona College of Medicine. Twelve-week-old C57BL/6 mice were treated with control TAT or AMPK-targeting peptide Pa496h (15 nmol/g/day) through intraperitoneal injection.

### *In vitro* phosphorylation assays

In experiments of AMPK autophosphorylation, purified FLAG-tagged AMPKα1β1γ1 or AMPKα2 β1γ1 (20 μM each) was incubated with AMPK inhibitor compound C (∼500 μM) in 1X kinase buffer (Cell Signaling, USA) in the presence or absence of 0.2 mM ATP at 37°C for 1 h.

For assessment of Pa2-491 peptide effect, purified AMPKα1 or α2 protein was incubated with Pa2-491 peptide at different concentrations (0, 0, 200, 400, 800 μM) plus 0.1 μg of PKA catalytic subunit (Millipore), 1X kinase buffer (Cell Signaling), and 0.2 mM ATP at room temperature for 30 min.

### Synthesis of AMPK targeting-peptides

AMPK targeting peptides Pa496h and Pa2-491 were synthesized at the Sequencing and Synthesis Facility of Johns Hopkins School of Medicine.

### Preparation of primary hepatocytes and glucose production assay

Primary hepatocytes were prepared from C57BL6 mice fed a chow diet or high-fat diet (60% calories from fat) following a protocol were published previously (28). After 24 h of seeding, primary hepatocytes were washed twice with PBS and subjected to serum starvation for 3 h. Then, the medium was replaced with 1 mL of glucose production buffer consisting of glucose-free DMEM supplemented with 20 mM sodium lactate and 2 mM sodium pyruvate or with 0.2 mM 8-bromo-cAMP. After 3 h of incubation, both medium and cells were collected.

### Confocal microscopy analysis

Primary hepatocytes were cultured in DMEM or treated with 100 μM of TAT, Pa496m, and Pa496h for 16 h, and then the medium was changed to DMEM without phenol red, supplemented with 50 nM MitoTracker^TM^ Red FM (M22425, Thermo Fisher Scientific, USA). Fluorescent images were acquired via a Zeiss confocal microscope (Zeiss Confocal LSM 880), as we reported previously (8). The excitation wavelengths of MitoTracker^TM^ Red CMR and Hoechst 33342 are at 561 and 405 nm, respectively.

### Statistical analysis

Statistical significance was calculated with a Student’s *t*-test and ANOVA test. Significance was accepted at the level of *P* < 0.05.

## Results

### AMPK activation leads to increased phosphorylation of catalytic α subunit at S496/S491

**Figure 1:**
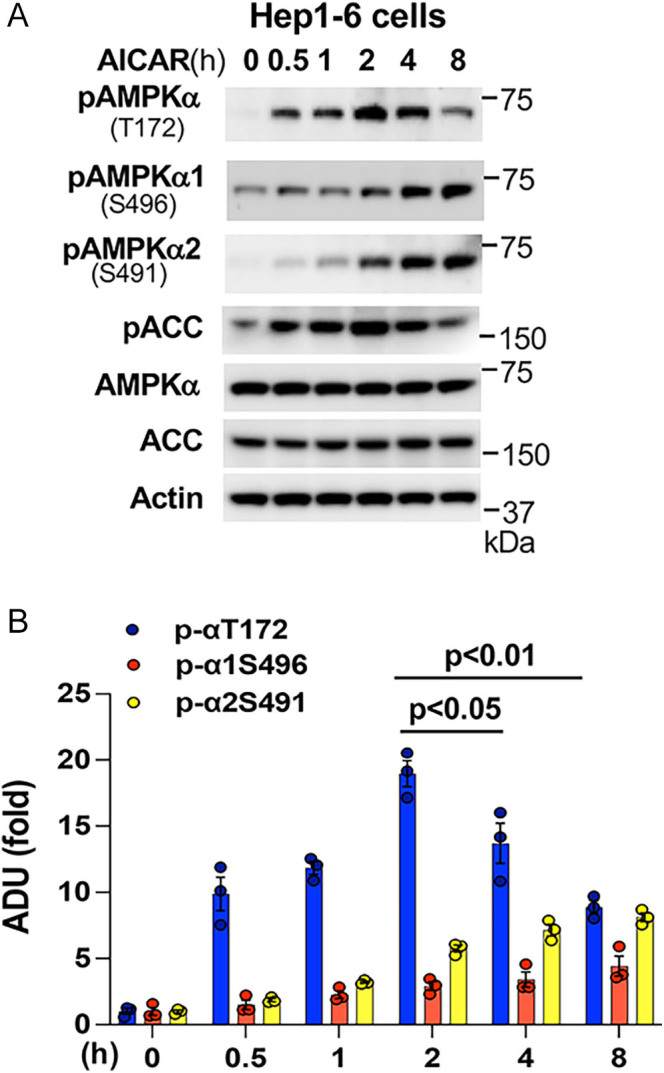
Activation of AMPK leads to autoinhibition by increasing phosphorylation of the catalytic α subunit at S496/S491. (A, B) Hep1-6 cells were treated with 1 mM AICAR for the indicated time (A), and densitometric analysis of phosphorylated AMPKα1/2 at T172, S496, and S491 (B) (*n* = 3). *, *P* < 0.05.

When we used the AMPK activator AICAR to treat Hep1-6 cells, we observed increased phosphorylation of AMPKα at T172 (a biomarker of AMPK activation) and phosphorylation of downstream target ACC after AICAR treatment for 1 h, indicating AMPK activation (Fig. 1A). However, of great interest, we also observed increased negative phosphorylation of the catalytic α subunit at S496/S491, along with decreased phosphorylation of AMPKα at T172 and downstream target ACC after AICAR treatment for 4 h (Fig. 1A and B). Noticeably, with prolonged phosphorylation of the catalytic α subunit at S496/S491, a greater reduction of phosphorylation of AMPKα at T172 and downstream target ACC was observed after AICAR treatment for 24 h. These data suggest that AMPK has a feedback inhibitory mechanism of governing its kinase activity.

### Autophosphorylation of AMPK catalytic α subunit at S496/S491

**Figure 2:**
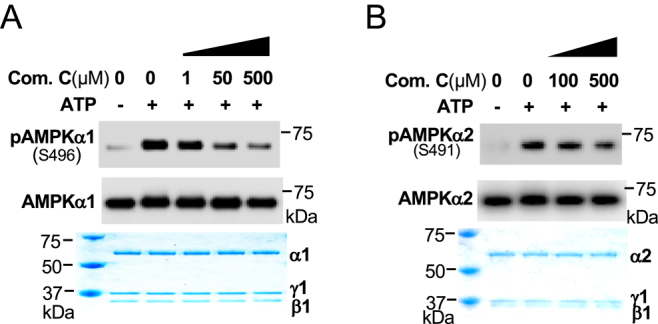
Functional AMPK complex directly phosphorylates the catalytic α subunit at S496/S491. (A, B) 20 μM of purified AMPKα1β1γ1 (A) or AMPKα2 β1γ1 (B) were incubated with indicated concentrations of AMPK inhibitor compound C at 37°C for 1 h.

To determine whether activation of AMPK can directly phosphorylate the catalytic α subunit at S496/S491 by itself, we used adenoviral expression vectors to express functional FLAG-tagged AMPKα1β1γ1 or AMPKα2β1γ1 and purified these heterotrimeric protein complexes (29). We then conducted an *in vitro* phosphorylation assay. Indeed, AMPKα1β1γ1 could phosphorylate the α1 subunit at S496, and inhibition of AMPK activity by Compound C decreased α1 subunit phosphorylation at S496 (Fig. 2A). Moreover, AMPKα2β1γ1 could phosphorylate the α2 subunit at S491 (Fig. 2B). These data confirmed that activation of AMPK can lead to the negative phosphorylation of the catalytic α subunit at S496/S491 by itself to maintain low AMPK activity.

### High concentrations of glucagon or insulin augment AMPKα1/2 phosphorylation at S496 and/or S491

**Figure 3:**
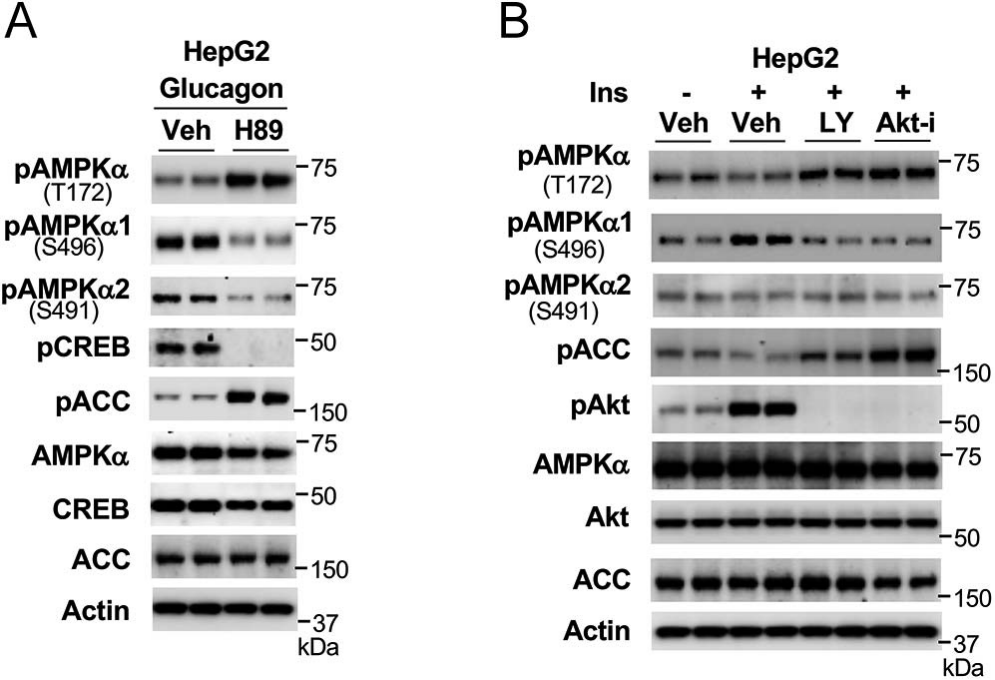
Activation of insulin signaling or glucagon signaling impairs AMPK activity. (A) HepG2 cells were treated with 20 μM H89 for 1 h, and then 50 nM glucagon for 3.5 h. (B) HepG2 cells were treated with 50 μM LY294002 or 25 μM Akt-i for 1 h, followed by the addition of 10 nM insulin for 4 h.

We reported that activation of cAMP-PKA signaling by hyperglucagonemia can augment AMPKα1 phosphorylation at S496 to impair AMPK activity in hepatocytes (22, 24). Furthermore, activation of the insulin signaling pathway by hyperinsulinemia can also augment AMPKα1 phosphorylation at S496 (25, 26, 27). Consistent with previous reports, in HepG2 cells, inhibition of PKA activity by inhibitor H89 blocked glucagon-stimulated AMPKα1 phosphorylation at S496, along with increased AMPK activity (increased phosphorylation of AMPKα at T172 and ACC) (Fig. 3A). Of note, inhibitor H89 also blocked AMPK α2 phosphorylation at S491, suggesting that activation of glucagon-cAMP-PKA signaling can augment AMPKα2 phosphorylation at S491 (Fig. 3A).

Treatment with insulin increased AMPKα1 phosphorylation at S496; in contrast, blockade of the PI3K-AKT signaling pathway by either PI3K inhibitor LY294002 or AKT inhibitor Akt-i decreased phosphorylation of AMPKα1 at S496, along with increased AMPKα phosphorylation at T172 (Fig. 3B). However, insulin treatment did not significantly change AMPKα2 phosphorylation at S491, suggesting that the impairment of AMPK activity by insulin occurs mainly through increasing phosphorylation of AMPKα1 at S496.

### Generation of AMPKα2S491 targeting-peptide to block α2 phosphorylation at S491

**Figure 4:**
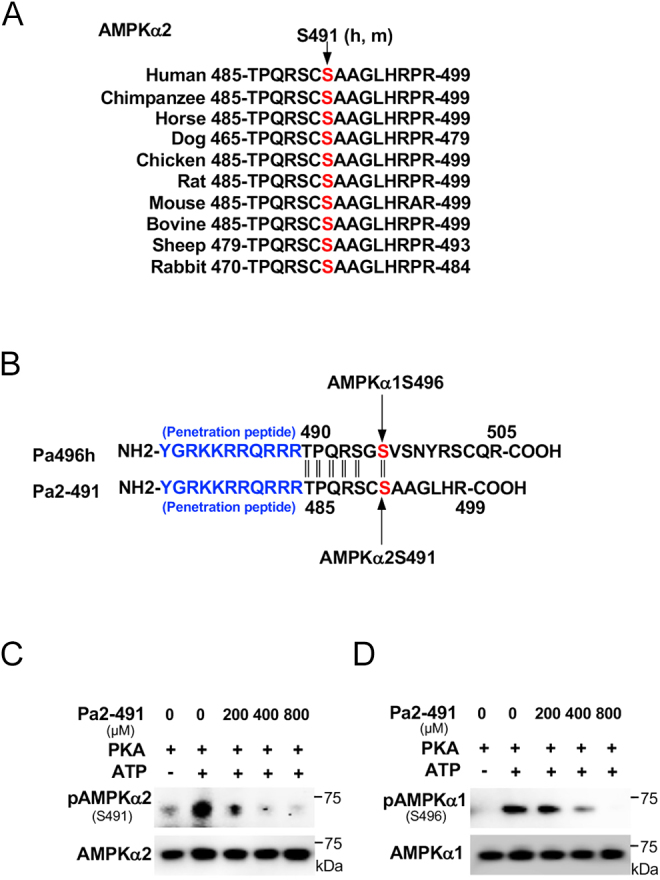
Generation of AMPKα2S491 targeting-peptide to block negative AMPK phosphorylation. (A) The phosphorylation site of S491 in AMPKα2 is conserved across species. (B) The amino acids of AMPK targeting-peptides Pa496h and Pa2-491. There are six same acids, including the serine phosphorylation site, at the N-terminal of AMPKa1 at S496 and AMPKa2 at S491. A cell penetration peptide TAT (blue color) was added at the N-terminal of each peptide. (C, D) Purified AMPKα2 (C) or AMPKα1 (D) protein (2 μM) was incubated with different concentrations (0, 0, 200, 400, 800 μM) of synthesized Pa2-491 peptide plus 0.1 μg of PKA catalytic subunit and incubated at room temperature for 30 min.

Previously, we designed and screened Pa496h (peptide anti-phosphorylation of AMPKα1S496, human) to competitively block/mask the phosphorylation of AMPKα1 at S496 by PKA or AKT (29). AMPK α1 and α2 subunits may have different functions in regulating cellular metabolism (4, 8), and the expression pattern of AMPKα1 and α2 subunits differs among tissues (11). Moreover, AMPKα2 S491 is conserved across species (Fig. 4A). Therefore, we designed and screened a new AMPKα2 targeting-peptide to block the phosphorylation of AMPKα2 at S491 (Fig. 4B). The cell-penetrating TAT sequence was added at the N-terminal of this α2-blocking peptide to generate a new AMPKα2 cellular targeting peptide (YGRKKRRQRRRTPQRSCSAAGLHR) and termed it Pa2-491 (peptide anti-phosphorylation of AMPKα2S491). Pa2-491 could block AMPKα2 phosphorylation at S491 (Fig. 4C) and AMPKα1 phosphorylation at S496 even to a lesser extent (Fig. 4D) by PKA in *in vitro* phosphorylation assays because of the five same amino acids at the N-terminus of the inhibitory phosphorylation site of AMPKα1 at S496 and AMPKα2 at S491 (Fig. 4B).

### Suppression of glucose production in hepatocytes by Pa2-491-activated AMPK

**Figure 5:**
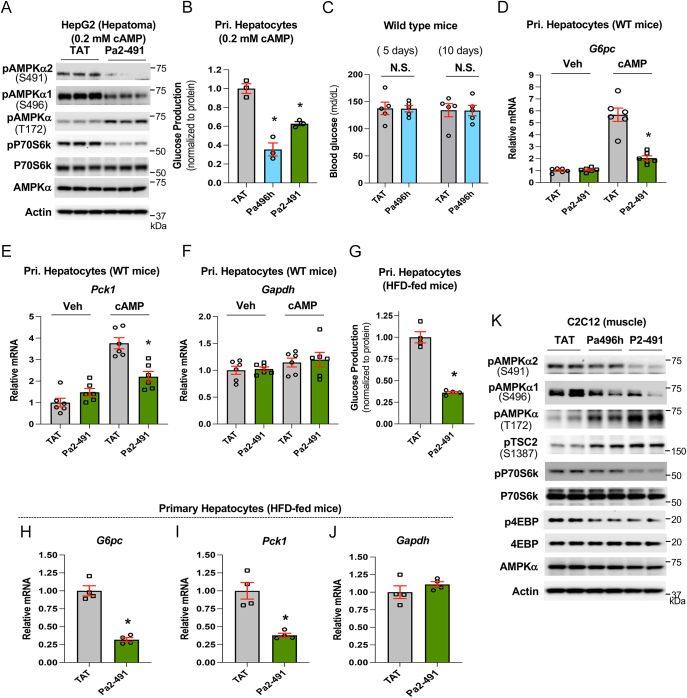
Activation of AMPK by Pa2-491 peptide. (A) Hepatoma HepG2 cells were treated with 100 μM of TAT control peptide, Pa496h, and Pa2-491 plus 0.2 mM cAMP for 16 h. (B) Primary hepatocytes prepared from WT mice were treated with 50 μM of Pa496h for 16 h, and then treated with Pa496h for 3 h during serum starvation, followed by incubation in glucose production medium supplemented with Pa496h and 0.2 mM Bt-cAMP (A) (*n* = 3). (C) 12-week-old WT mice were treated with Pa496h (15 nmol/g/day, ∼10-day). Blood glucose levels (6 h fast, *n* = 5). (D, E, F) Primary hepatocytes from WT mice were treated with Pa2-491 as in B with or without Bt-cAMP (*n* = 6). (G, H, I, J) Primary hepatocytes prepared from high-fat-diet (12 weeks)-fed mice were treated with 50 μM Pa2-491 (16 h), then treated with Pa2-491 for 3 h during serum starvation, followed by incubation in glucose production medium supplemented with Pa2-419 for 3 h (G). Another set of treatment was conducted for determining mRNA levels of *G6pc* (H), *Pck1* (I), and *Gapdh* (J) (*n* = 4). (K) C2C12 cells were treated with 100 μM of TAT control peptide, Pa496h, and Pa2-491 for 16 h.

Hepatocytes have both AMPK catalytic subunits. We used the Pa2-491 peptide to treat hepatoma HepG2 cells with activated cAMP-PKA signaling and found that Pa2-491 treatment significantly blocked phosphorylation of AMPKα2 at S491 and α1 at S496, along with increased AMPKαT172 phosphorylation (Fig. 5A). In primary hepatocytes prepared from wild-type mice, both Pa496h and Pa2-491 could significantly inhibit cAMP-stimulated glucose production (Fig. 5B). Since hyperglucagonemia and/or hyperinsulinemia can augment AMPKα1/2 phosphorylation at S496/491 to impair AMPK activity and regulation of liver glucose production, blocking AMPKα1/2 phosphorylation at S496/491 by our targeting peptides Pa496h or Pa496m (mouse form) could significantly improve hyperglycemia in obese mice (29). Because Pa496h has a greater effect than Pa2-491 on suppression of glucose production in hepatocytes (Fig. 5B), we employed Pa496h to treat wild-type mice fed a *chow* diet and found that Pa496h had no effect on fasting blood levels (Fig. 5C), as it did in obese mice (29). This is due to the low AMPKα1/2 phosphorylation at S496/491 in healthy mice (29). Consistent with this notion, in primary hepatocytes prepared from wild-mice, treatment with Pa2-491 had no significant impact on the expression of rate-limiting gluconeogenic genes, *G6pc* and *Pck1*. In contrast, Pa2-491 significantly suppressed *G6pc* and *Pck1* gene expression in primary hepatocytes treated with cAMP (Fig. 5D, E, F).

Furthermore, in primary hepatocytes prepared from high-fat-diet (12 weeks)-fed mice, treatment with Pa2-491 significantly suppressed glucose production and *G6pc* and *Pck1* gene expression (Fig. 5G, H, I, J). To verify further that Pa2-491 can block the negative phosphorylation of AMPKα1/2 at S496/491 in cells other than hepatocytes; we treated C2C12 muscle cells, as AMPKα2 is the principal catalytic subunit in muscle (11). Compared to Pa496h treatment, Pa2-491 treatment had a greater effect on blocking AMPKα1/2 at S496/491 and AMPK activation in C2C12 cells (Fig. 5K).

### Promotion of mitochondrial dynamics by Pa2-491 peptide

**Figure 6:**
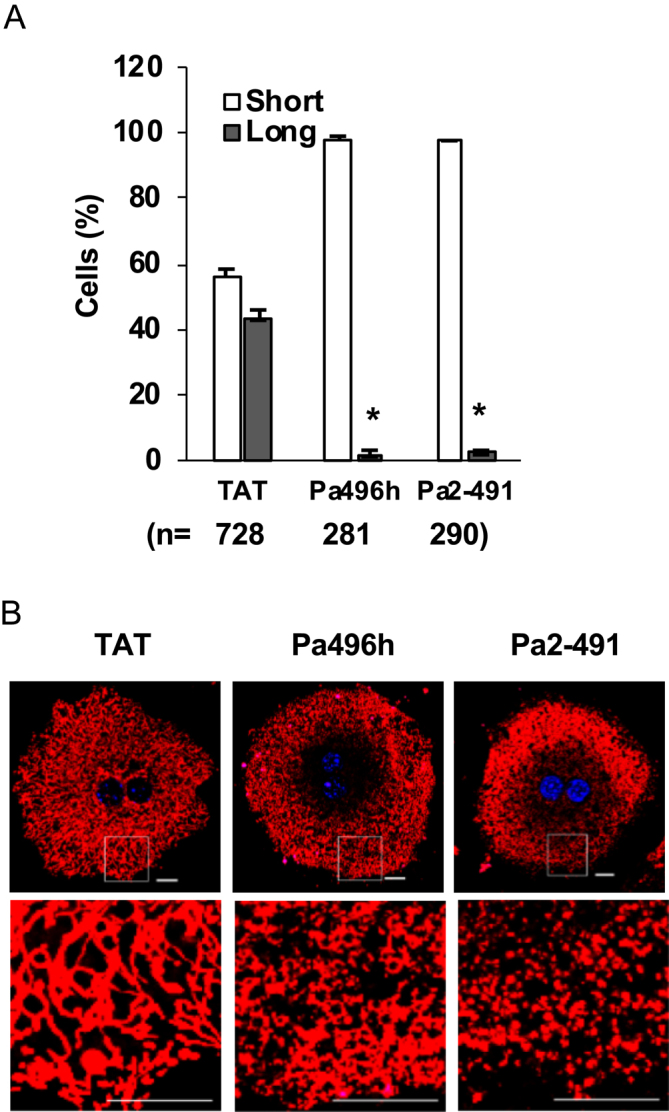
Activation of AMPK by Pa2-491 peptide promotes mitochondrial fission in hepatocytes. (A, B) Primary hepatocytes were isolated from 6-month-old mice, and treated with 100 μM of Pa496h or Pa2-491 for 16 h, and mitochondria were stained with MitoTracker Red, then short and elongated mitochondria were counted.

Mitochondria continually undergo fusion and fission, and compromised mitochondria are eliminated via mitophagy after fission (30, 31, 32, 33, 34). In hepatocytes of obese patients and aged individuals, mitochondria become elongated (35, 36, 37) or form megamitochondria (38, 39, 40). Our previous report showed that the AMPKα1 targeting peptide Pa496h/m can promote mitochondrial fission to allow the elimination of compromised mitochondria through mitophagy, a process following mitochondrial fission (29). We determined the effect of the Pa2-491 peptide on mitochondrial dynamics in primary hepatocytes prepared from 6-month-old mice and found that treatment with the Pa2-491 peptide drastically reduced elongated mitochondria to a similar extent as Pa496h (Fig. 6A and B).

## Discussion

AMPK is a master regulator of metabolism, playing a key role in regulating metabolism and maintaining cellular energy homeostasis. Phosphorylation of α subunits at T172 by upstream kinases, such as the tumor-suppressor liver kinase B1 (LKB1), has a critical role in the activation of enzyme activity (41). The β subunits have no enzymatic activity; however, they function as a scaffold protein for AMPK αβγ complex assembly. The γ subunits can bind adenine nucleotides because of four cystathionine-beta-synthase (CBS) domains. When cellular energy status is falling, with increased AMP/ATP or ADP/ATP ratio, this results in a change in nucleotide binding and an allosteric change in AMPK (5), leading to an increase in net phosphorylation of the α subunit at T172 and AMPK activation.

It is not well understood how the molecular mechanisms maintain low AMPK activity in unstressed cells or restrict it from overactivation. We and other investigators found that glucagon-activated PKA (22, 42) or insulin-activated AKT (25, 26, 27) directly phosphorylates AMPKα1/2 at S496/491 to impair AMPK activity. Furthermore, high glucose levels also increase AMPKα1 phosphorylation at S496 (43), suggesting that high nutrient conditions can suppress AMPK activity by increasing AMPKα1/2 phosphorylation at S496/491. Of great interest, when we used the AMPK activator AICAR to treat hepatocytes, we found that activation of AMPK (increased AMPKα phosphorylation at T172) was followed by elevated and prolonged phosphorylation of AMPKα1/2 at S496/S491, suggesting feedback limiting AMPK activity. Indeed, in an *in vitro* phosphorylation assay, functional AMPK αβγ can phosphorylate AMPKα1/2 at S496/S491, revealing a mechanism of self-inhibition of AMPK activity. This self-inhibition mechanism will restrain AMPK from overactivation and timely adjust cellular energy status as needed.

AMPK α1 and α2 subunits may have different functions in regulating cellular metabolism (4, 8), and the expression pattern of AMPK α1 and α2 subunits differs among tissues (11). We therefore generated a new AMPKα2 targeting-peptide, Pa2-491, to block the negative phosphorylation at S491. This peptide can effectively block AMPKα2 phosphorylation at S491 to activate AMPK, resulting in the suppression of gluconeogenic gene expression and glucose production in hepatocytes, and promotion of mitochondrial fission. Because of insulin resistance (the hallmark of T2DM and obesity), increased liver glucose production is a major cause of hyperglycemia in patients with T2DM and obesity (44, 45, 46). Activation of AMPK suppresses glucose production in the liver and improves hyperglycemia in these patients (11, 12, 22, 47); therefore, AMPK is an ideal therapeutic target for treating T2DM and obesity. However, to date, no direct AMPK activator is available in the clinic. This phenomenon may be due to: i) activated AKT by hyperinsulinemia (48, 49), ii) activated PKA (22, 42) by hyperglucagonemia (50, 51, 52), iii) hyperglycemia (43), and iv) activated AMPKα1/2 increasing the negative AMPKα1/2 phosphorylation at S496/491 to inhibit AMPK activity. Thus, AMPK targeting-peptide Pa496h and Pa2-491 peptide can potentially be used to activate AMPK in obesity and T2DM. In particular, Pa2-491 peptide can be used to activate AMPK in tissue, such as muscle, in which AMPKα2 is the dominant catalytic subunit.

## Declaration of interest

The authors declare that there is no conflict of interest that could be perceived as prejudicing the impartiality of the work reported.

## Funding

This work was supported in part by grants from the National Institute of Diabetes and Digestive and Kidney Diseaseshttps://doi.org/10.13039/100000062: R01DK120309 and R01DK107641.
